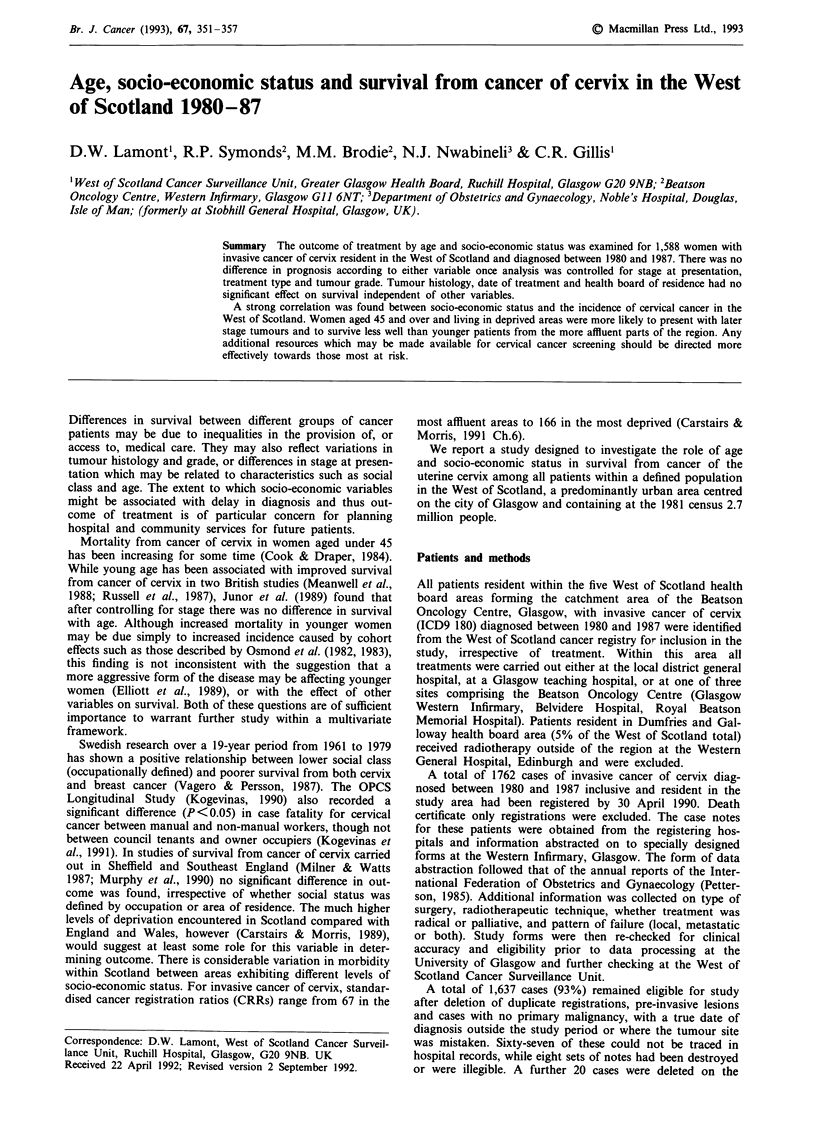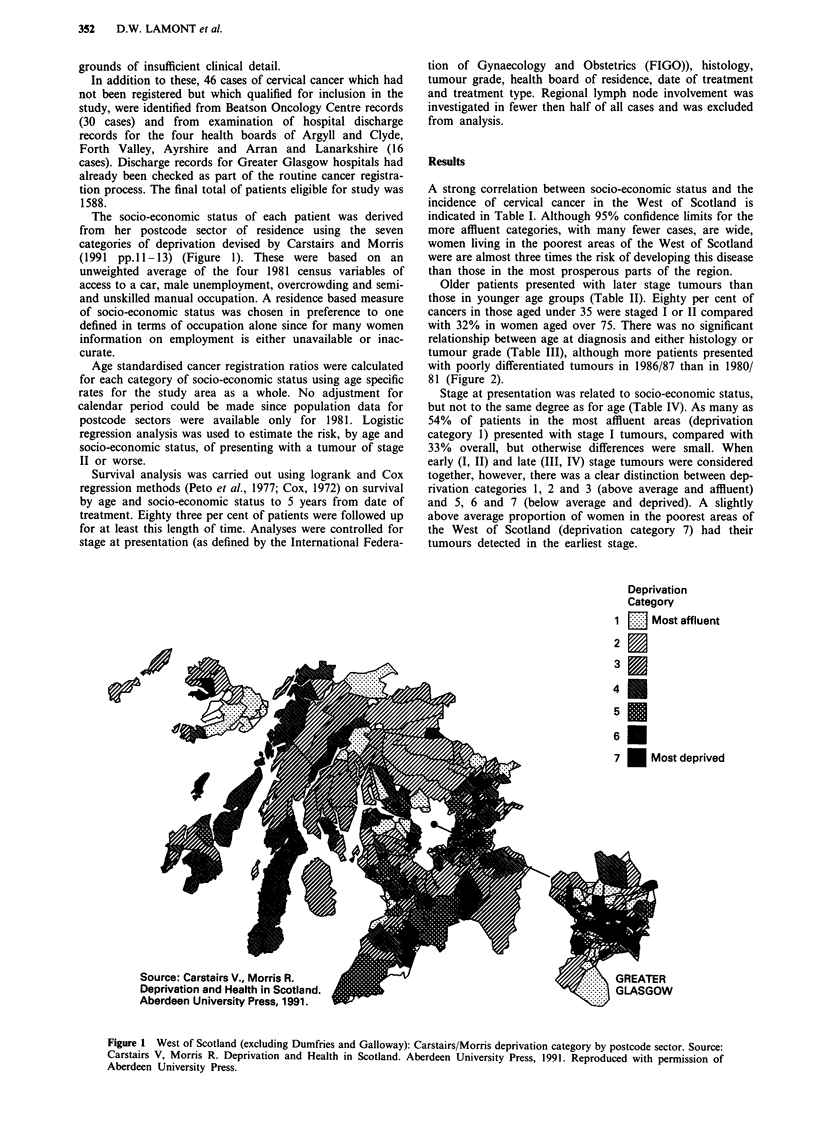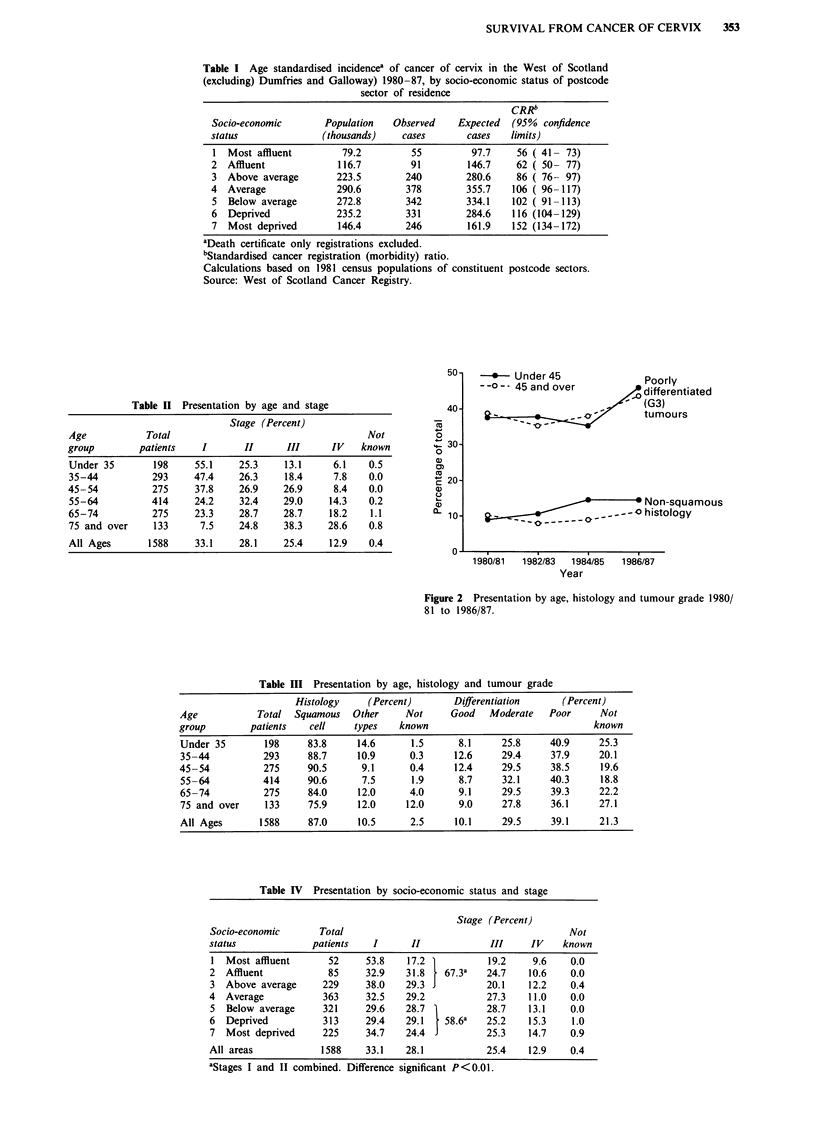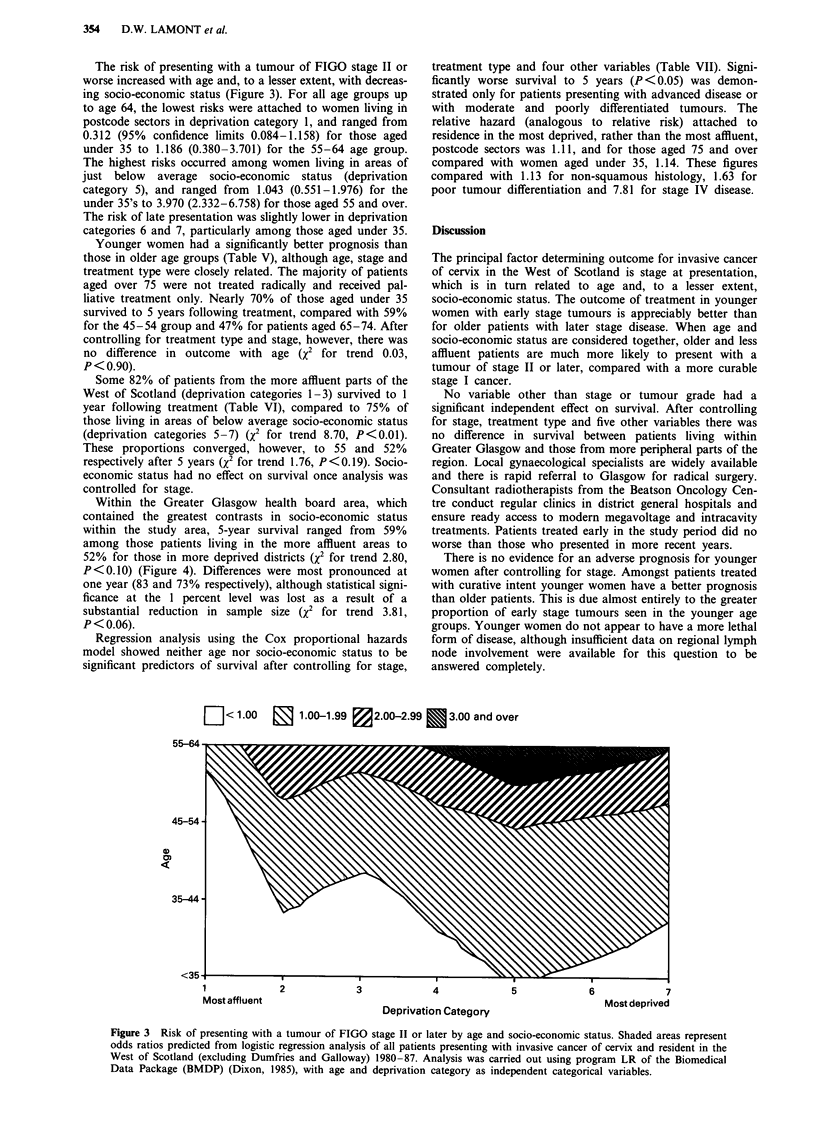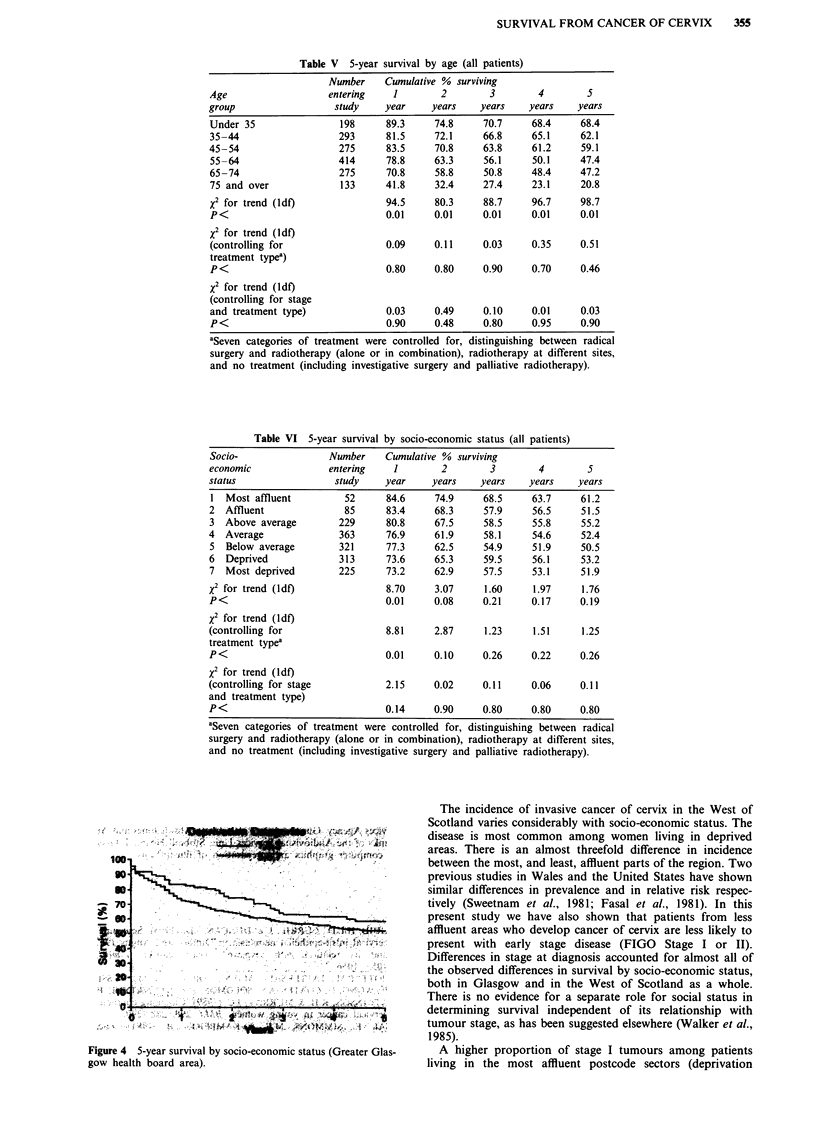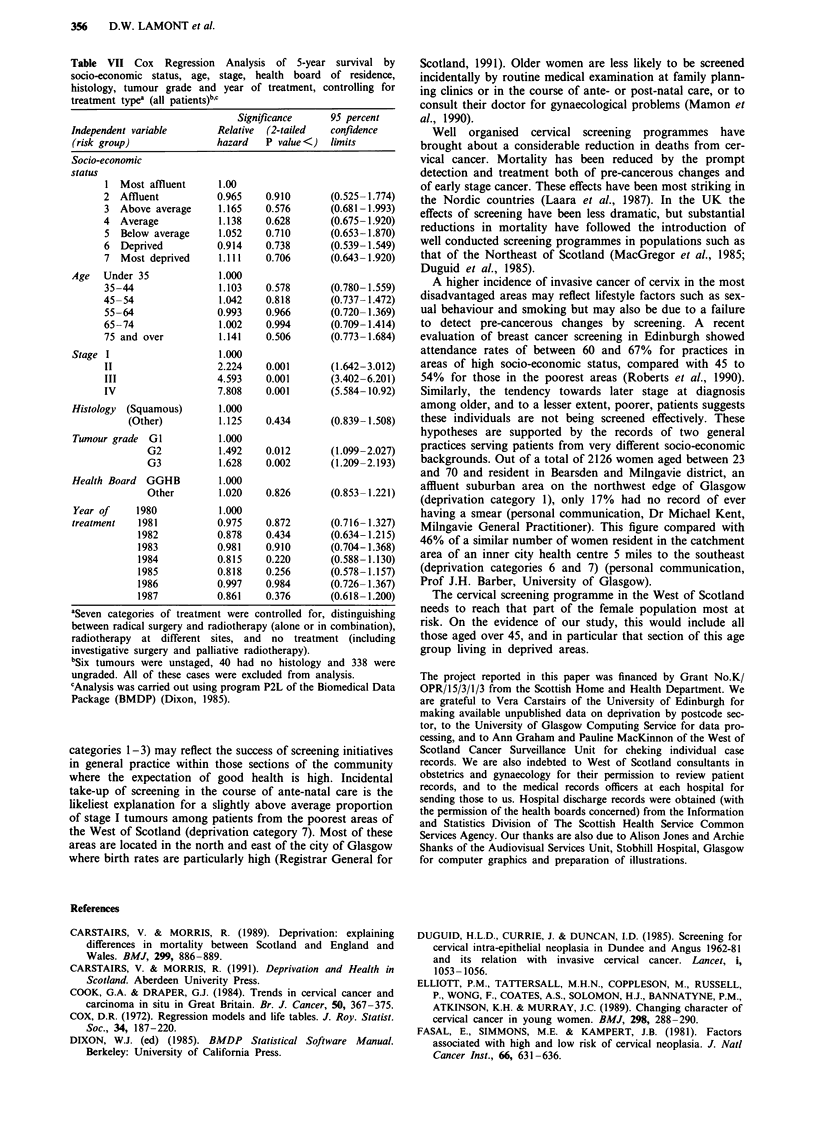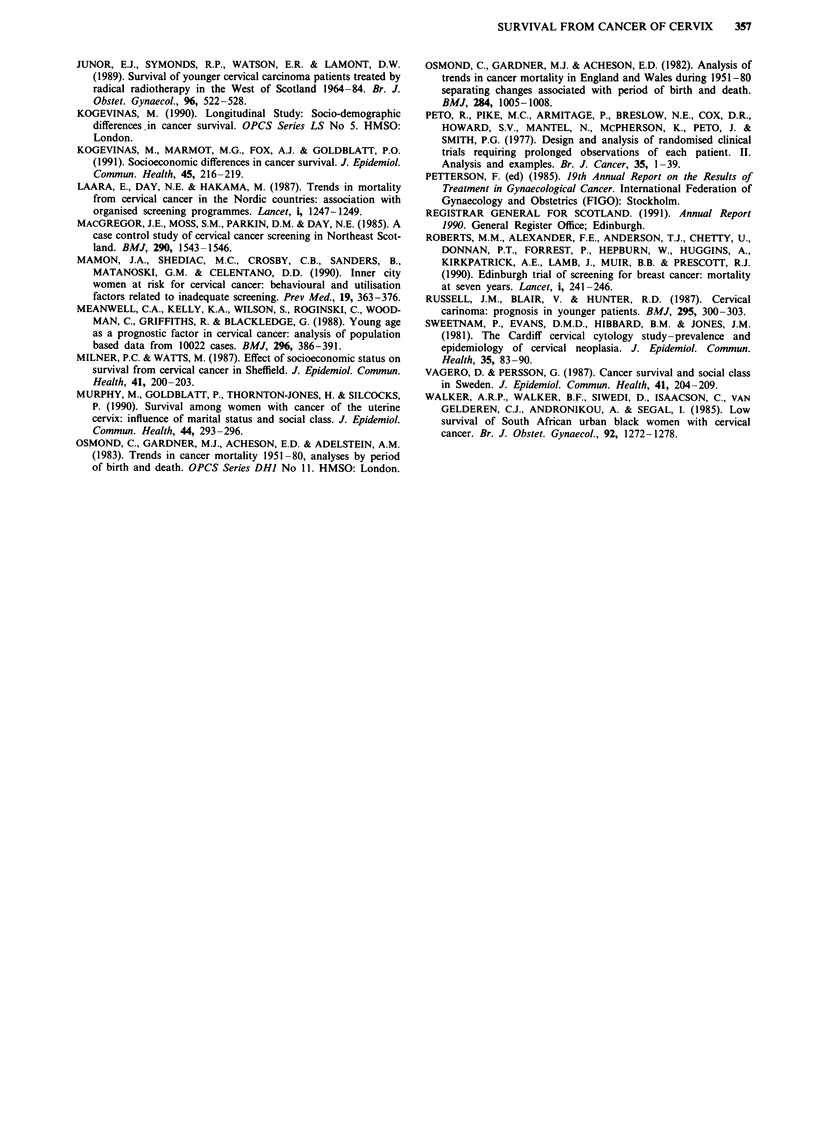# Age, socio-economic status and survival from cancer of cervix in the West of Scotland 1980-87.

**DOI:** 10.1038/bjc.1993.64

**Published:** 1993-02

**Authors:** D. W. Lamont, R. P. Symonds, M. M. Brodie, N. J. Nwabineli, C. R. Gillis

**Affiliations:** West of Scotland Cancer Surveillance Unit, Greater Glasgow Health Board, Ruchill Hospital, UK.

## Abstract

The outcome of treatment by age and socio-economic status was examined for 1,588 women with invasive cancer of cervix resident in the West of Scotland and diagnosed between 1980 and 1987. There was no difference in prognosis according to either variable once analysis was controlled for stage at presentation, treatment type and tumour grade. Tumour histology, date of treatment and health board of residence had no significant effect on survival independent of other variables. A strong correlation was found between socio-economic status and the incidence of cervical cancer in the West of Scotland. Women aged 45 and over and living in deprived areas were more likely to present with later stage tumours and to survive less well than younger patients from the more affluent parts of the region. Any additional resources which may be made available for cervical cancer screening should be directed more effectively towards those most at risk.


Br. J. Cancer (1993), 67, 351-357 © Macmillan Press Ltd., 1993

### Age, socio-economic status and survival from cancer of cervix in the West of Scotland 1980-87

D.W. Lamont[1], R.P. Symonds[2], M.M. Brodie[2], N.J. Nwabineli[3] & C.R. Gillis[1]

[1]*West of Scotland Cancer Surveillance Unit, Greater Glasgow Health Board, Ruchill Hospital, Glasgow G20 9NB;* [2]*Beatson Oncology Centre, Western Infirmary, Glasgow G11 6NT;* [3]*Department of Obstetrics and Gynaecology, Noble's Hospital, Douglas, Isle of Man; (formerly at Stobhill General Hospital, Glasgow, UK).*

**Summary** The outcome of treatment by age and socio-economic status was examined for 1,588 women with invasive cancer of cervix resident in the West of Scotland and diagnosed between 1980 and 1987. There was no difference in prognosis according to either variable once analysis was controlled for stage at presentation, treatment type and tumour grade. Tumour histology, date of treatment and health board of residence had no significant effect on survival independent of other variables.

A strong correlation was found between socio-economic status and the incidence of cervical cancer in the West of Scotland. Women aged 45 and over and living in deprived areas were more likely to present with later stage tumours and to survive less well than younger patients from the more affluent parts of the region. Any additional resources which may be made available for cervical cancer screening should be directed more effectively towards those most at risk.

Differences in survival between different groups of cancer patients may be due to inequalities in the provision of, or access to, medical care. They may also reflect variations in tumour histology and grade, or differences in stage at presentation which may be related to characteristics such as social class and age. The extent to which socio-economic variables might be associated with delay in diagnosis and thus outcome of treatment is of particular concern for planning hospital and community services for future patients.

Mortality from cancer of cervix in women aged under 45 has been increasing for some time (Cook & Draper, 1984). While young age has been associated with improved survival from cancer of cervix in two British studies (Meanwell *et al.*, 1988; Russell *et al.*, 1987), Junor *et al.* (1989) found that after controlling for stage there was no difference in survival with age. Although increased mortality in younger women may be due simply to increased incidence caused by cohort effects such as those described by Osmond *et al.* (1982, 1983), this finding is not inconsistent with the suggestion that a more aggressive form of the disease may be affecting younger women (Elliott *et al.*, 1989), or with the effect of other variables on survival. Both of these questions are of sufficient importance to warrant further study within a multivariate framework.

Swedish research over a 19-year period from 1961 to 1979 has shown a positive relationship between lower social class (occupationally defined) and poorer survival from both cervix and breast cancer (Vagero & Persson, 1987). The OPCS Longitudinal Study (Kogevinas, 1990) also recorded a significant difference ($P<0.05$) in case fatality for cervical cancer between manual and non-manual workers, though not between council tenants and owner occupiers (Kogevinas *et al.*, 1991). In studies of survival from cancer of cervix carried out in Sheffield and Southeast England (Milner & Watts 1987; Murphy *et al.*, 1990) no significant difference in outcome was found, irrespective of whether social status was defined by occupation or area of residence. The much higher levels of deprivation encountered in Scotland compared with England and Wales, however (Carstairs & Morris, 1989), would suggest at least some role for this variable in determining outcome. There is considerable variation in morbidity within Scotland between areas exhibiting different levels of socio-economic status. For invasive cancer of cervix, standardised cancer registration ratios (CRRs) range from 67 in the most affluent areas to 166 in the most deprived (Carstairs & Morris, 1991 Ch.6).

We report a study designed to investigate the role of age and socio-economic status in survival from cancer of the uterine cervix among all patients within a defined population in the West of Scotland, a predominantly urban area centred on the city of Glasgow and containing at the 1981 census 2.7 million people.

### Patients and methods

All patients resident within the five West of Scotland health board areas forming the catchment area of the Beatson Oncology Centre, Glasgow, with invasive cancer of cervix (ICD9 180) diagnosed between 1980 and 1987 were identified from the West of Scotland cancer registry for inclusion in the study, irrespective of treatment. Within this area all treatments were carried out either at the local district general hospital, at a Glasgow teaching hospital, or at one of three sites comprising the Beatson Oncology Centre (Glasgow Western Infirmary, Belvidere Hospital, Royal Beatson Memorial Hospital). Patients resident in Dumfries and Galloway health board area (5% of the West of Scotland total) received radiotherapy outside of the region at the Western General Hospital, Edinburgh and were excluded.

A total of 1762 cases of invasive cancer of cervix diagnosed between 1980 and 1987 inclusive and resident in the study area had been registered by 30 April 1990. Death certificate only registrations were excluded. The case notes for these patients were obtained from the registering hospitals and information abstracted on to specially designed forms at the Western Infirmary, Glasgow. The form of data abstraction followed that of the annual reports of the International Federation of Obstetrics and Gynaecology (Petterson, 1985). Additional information was collected on type of surgery, radiotherapeutic technique, whether treatment was radical or palliative, and pattern of failure (local, metastatic or both). Study forms were then re-checked for clinical accuracy and eligibility prior to data processing at the University of Glasgow and further checking at the West of Scotland Cancer Surveillance Unit.

A total of 1,637 cases (93%) remained eligible for study after deletion of duplicate registrations, pre-invasive lesions and cases with no primary malignancy, with a true date of diagnosis outside the study period or where the tumour site was mistaken. Sixty-seven of these could not be traced in hospital records, while eight sets of notes had been destroyed or were illegible. A further 20 cases were deleted on the

Correspondence: D.W. Lamont, West of Scotland Cancer Surveillance Unit, Ruchill Hospital, Glasgow, G20 9NB. UK
Received 22 April 1992; Revised version 2 September 1992.

grounds of insufficient clinical detail.

In addition to these, 46 cases of cervical cancer which had not been registered but which qualified for inclusion in the study, were identified from Beatson Oncology Centre records (30 cases) and from examination of hospital discharge records for the four health boards of Argyll and Clyde, Forth Valley, Ayrshire and Arran and Lanarkshire (16 cases). Discharge records for Greater Glasgow hospitals had already been checked as part of the routine cancer registration process. The final total of patients eligible for study was 1588.

The socio-economic status of each patient was derived from her postcode sector of residence using the seven categories of deprivation devised by Carstairs and Morris (1991 pp.11–13) (Figure 1). These were based on an unweighted average of the four 1981 census variables of access to a car, male unemployment, overcrowding and semi- and unskilled manual occupation. A residence based measure of socio-economic status was chosen in preference to one defined in terms of occupation alone since for many women information on employment is either unavailable or inaccurate.

Age standardised cancer registration ratios were calculated for each category of socio-economic status using age specific rates for the study area as a whole. No adjustment for calendar period could be made since population data for postcode sectors were available only for 1981. Logistic regression analysis was used to estimate the risk, by age and socio-economic status, of presenting with a tumour of stage II or worse.

Survival analysis was carried out using logrank and Cox regression methods (Peto *et al.*, 1977; Cox, 1972) on survival by age and socio-economic status to 5 years from date of treatment. Eighty three per cent of patients were followed up for at least this length of time. Analyses were controlled for stage at presentation (as defined by the International Federation of Gynaecology and Obstetrics (FIGO)), histology, tumour grade, health board of residence, date of treatment and treatment type. Regional lymph node involvement was investigated in fewer then half of all cases and was excluded from analysis.

### Results

A strong correlation between socio-economic status and the incidence of cervical cancer in the West of Scotland is indicated in Table I. Although 95% confidence limits for the more affluent categories, with many fewer cases, are wide, women living in the poorest areas of the West of Scotland were are almost three times the risk of developing this disease than those in the most prosperous parts of the region.

Older patients presented with later stage tumours than those in younger age groups (Table II). Eighty per cent of cancers in those aged under 35 were staged I or II compared with 32% in women aged over 75. There was no significant relationship between age at diagnosis and either histology or tumour grade (Table III), although more patients presented with poorly differentiated tumours in 1986/87 than in 1980/81 (Figure 2).

Stage at presentation was related to socio-economic status, but not to the same degree as for age (Table IV). As many as 54% of patients in the most affluent areas (deprivation category 1) presented with stage I tumours, compared with 33% overall, but otherwise differences were small. When early (I, II) and late (III, IV) stage tumours were considered together, however, there was a clear distinction between deprivation categories 1, 2 and 3 (above average and affluent) and 5, 6 and 7 (below average and deprived). A slightly above average proportion of women in the poorest areas of the West of Scotland (deprivation category 7) had their tumours detected in the earliest stage.

**Figure 1** West of Scotland (excluding Dumfries and Galloway): Carstairs/Morris deprivation category by postcode sector. Source: Carstairs V, Morris R. Deprivation and Health in Scotland. Aberdeen University Press, 1991. Reproduced with permission of Aberdeen University Press.

**Table I** Age standardised incidence[a] of cancer of cervix in the West of Scotland (excluding) Dumfries and Galloway) 1980–87, by socio-economic status of postcode sector of residence

| *Socio-economic status* | *Population (thousands)* | *Observed cases* | *Expected cases* | *CRR*[b] *(95% confidence limits)* |
|---|---|---|---|---|
| 1 Most affluent | 79.2 | 55 | 97.7 | 56 ( 41– 73) |
| 2 Affluent | 116.7 | 91 | 146.7 | 62 ( 50– 77) |
| 3 Above average | 223.5 | 240 | 280.6 | 86 ( 76– 97) |
| 4 Average | 290.6 | 378 | 355.7 | 106 ( 96–117) |
| 5 Below average | 272.8 | 342 | 334.1 | 102 ( 91–113) |
| 6 Deprived | 235.2 | 331 | 284.6 | 116 (104–129) |
| 7 Most deprived | 146.4 | 246 | 161.9 | 152 (134–172) |

[a]Death certificate only registrations excluded.
[b]Standardised cancer registration (morbidity) ratio.
Calculations based on 1981 census populations of constituent postcode sectors.
Source: West of Scotland Cancer Registry.

**Table II** Presentation by age and stage

| *Age group* | *Total patients* | *Stage (Percent)* *I* | *II* | *III* | *IV* | *Not known* |
|---|---|---|---|---|---|---|
| Under 35 | 198 | 55.1 | 25.3 | 13.1 | 6.1 | 0.5 |
| 35–44 | 293 | 47.4 | 26.3 | 18.4 | 7.8 | 0.0 |
| 45–54 | 275 | 37.8 | 26.9 | 26.9 | 8.4 | 0.0 |
| 55–64 | 414 | 24.2 | 32.4 | 29.0 | 14.3 | 0.2 |
| 65–74 | 275 | 23.3 | 28.7 | 28.7 | 18.2 | 1.1 |
| 75 and over | 133 | 7.5 | 24.8 | 38.3 | 28.6 | 0.8 |
| All Ages | 1588 | 33.1 | 28.1 | 25.4 | 12.9 | 0.4 |

**Figure 2** Presentation by age, histology and tumour grade 1980/81 to 1986/87.

**Table III** Presentation by age, histology and tumour grade

| *Age group* | *Total patients* | *Histology (Percent)* *Squamous cell* | *Other types* | *Not known* | *Differentiation (Percent)* *Good* | *Moderate* | *Poor* | *Not known* |
|---|---|---|---|---|---|---|---|---|
| Under 35 | 198 | 83.8 | 14.6 | 1.5 | 8.1 | 25.8 | 40.9 | 25.3 |
| 35–44 | 293 | 88.7 | 10.9 | 0.3 | 12.6 | 29.4 | 37.9 | 20.1 |
| 45–54 | 275 | 90.5 | 9.1 | 0.4 | 12.4 | 29.5 | 38.5 | 19.6 |
| 55–64 | 414 | 90.6 | 7.5 | 1.9 | 8.7 | 32.1 | 40.3 | 18.8 |
| 65–74 | 275 | 84.0 | 12.0 | 4.0 | 9.1 | 29.5 | 39.3 | 22.2 |
| 75 and over | 133 | 75.9 | 12.0 | 12.0 | 9.0 | 27.8 | 36.1 | 27.1 |
| All Ages | 1588 | 87.0 | 10.5 | 2.5 | 10.1 | 29.5 | 39.1 | 21.3 |

**Table IV** Presentation by socio-economic status and stage

| *Socio-economic status* | *Total patients* | *Stage (Percent)* *I* | *II* | | *III* | *IV* | *Not known* |
|---|---|---|---|---|---|---|---|
| 1 Most affluent | 52 | 53.8 | 17.2 | 67.3[a] | 19.2 | 9.6 | 0.0 |
| 2 Affluent | 85 | 32.9 | 31.8 | | 24.7 | 10.6 | 0.0 |
| 3 Above average | 229 | 38.0 | 29.3 | | 20.1 | 12.2 | 0.4 |
| 4 Average | 363 | 32.5 | 29.2 | | 27.3 | 11.0 | 0.0 |
| 5 Below average | 321 | 29.6 | 28.7 | 58.6[a] | 28.7 | 13.1 | 0.0 |
| 6 Deprived | 313 | 29.4 | 29.1 | | 25.2 | 15.3 | 1.0 |
| 7 Most deprived | 225 | 34.7 | 24.4 | | 25.3 | 14.7 | 0.9 |
| All areas | 1588 | 33.1 | 28.1 | | 25.4 | 12.9 | 0.4 |

[a]Stages I and II combined. Difference significant $P<0.01$.

The risk of presenting with a tumour of FIGO stage II or worse increased with age and, to a lesser extent, with decreasing socio-economic status (Figure 3). For all age groups up to age 64, the lowest risks were attached to women living in postcode sectors in deprivation category 1, and ranged from 0.312 (95% confidence limits 0.084–1.158) for those aged under 35 to 1.186 (0.380–3.701) for the 55–64 age group. The highest risks occurred among women living in areas of just below average socio-economic status (deprivation category 5), and ranged from 1.043 (0.551–1.976) for the under 35's to 3.970 (2.332–6.758) for those aged 55 and over. The risk of late presentation was slightly lower in deprivation categories 6 and 7, particularly among those aged under 35.

Younger women had a significantly better prognosis than those in older age groups (Table V), although age, stage and treatment type were closely related. The majority of patients aged over 75 were not treated radically and received palliative treatment only. Nearly 70% of those aged under 35 survived to 5 years following treatment, compared with 59% for the 45–54 group and 47% for patients aged 65–74. After controlling for treatment type and stage, however, there was no difference in outcome with age ($\chi^2$ for trend 0.03, $P<0.90$).

Some 82% of patients from the more affluent parts of the West of Scotland (deprivation categories 1–3) survived to 1 year following treatment (Table VI), compared to 75% of those living in areas of below average socio-economic status (deprivation categories 5–7) ($\chi^2$ for trend 8.70, $P<0.01$). These proportions converged, however, to 55 and 52% respectively after 5 years ($\chi^2$ for trend 1.76, $P<0.19$). Socio-economic status had no effect on survival once analysis was controlled for stage.

Within the Greater Glasgow health board area, which contained the greatest contrasts in socio-economic status within the study area, 5-year survival ranged from 59% among those patients living in the more affluent areas to 52% for those in more deprived districts ($\chi^2$ for trend 2.80, $P<0.10$) (Figure 4). Differences were most pronounced at one year (83 and 73% respectively), although statistical significance at the 1 percent level was lost as a result of a substantial reduction in sample size ($\chi^2$ for trend 3.81, $P<0.06$).

Regression analysis using the Cox proportional hazards model showed neither age nor socio-economic status to be significant predictors of survival after controlling for stage, treatment type and four other variables (Table VII). Significantly worse survival to 5 years ($P<0.05$) was demonstrated only for patients presenting with advanced disease or with moderate and poorly differentiated tumours. The relative hazard (analogous to relative risk) attached to residence in the most deprived, rather than the most affluent, postcode sectors was 1.11, and for those aged 75 and over compared with women aged under 35, 1.14. These figures compared with 1.13 for non-squamous histology, 1.63 for poor tumour differentiation and 7.81 for stage IV disease.

### Discussion

The principal factor determining outcome for invasive cancer of cervix in the West of Scotland is stage at presentation, which is in turn related to age and, to a lesser extent, socio-economic status. The outcome of treatment in younger women with early stage tumours is appreciably better than for older patients with later stage disease. When age and socio-economic status are considered together, older and less affluent patients are much more likely to present with a tumour of stage II or later, compared with a more curable stage I cancer.

No variable other than stage or tumour grade had a significant independent effect on survival. After controlling for stage, treatment type and five other variables there was no difference in survival between patients living within Greater Glasgow and those from more peripheral parts of the region. Local gynaecological specialists are widely available and there is rapid referral to Glasgow for radical surgery. Consultant radiotherapists from the Beatson Oncology Centre conduct regular clinics in district general hospitals and ensure ready access to modern megavoltage and intracavity treatments. Patients treated early in the study period did no worse than those who presented in more recent years.

There is no evidence for an adverse prognosis for younger women after controlling for stage. Amongst patients treated with curative intent younger women have a better prognosis than older patients. This is due almost entirely to the greater proportion of early stage tumours seen in the younger age groups. Younger women do not appear to have a more lethal form of disease, although insufficient data on regional lymph node involvement were available for this question to be answered completely.

**Figure 3** Risk of presenting with a tumour of FIGO stage II or later by age and socio-economic status. Shaded areas represent odds ratios predicted from logistic regression analysis of all patients presenting with invasive cancer of cervix and resident in the West of Scotland (excluding Dumfries and Galloway) 1980–87. Analysis was carried out using program LR of the Biomedical Data Package (BMDP) (Dixon, 1985), with age and deprivation category as independent categorical variables.

**Table V** 5-year survival by age (all patients)

| *Age group* | *Number entering study* | *Cumulative % surviving 1 year* | *2 years* | *3 years* | *4 years* | *5 years* |
|---|---|---|---|---|---|---|
| Under 35 | 198 | 89.3 | 74.8 | 70.7 | 68.4 | 68.4 |
| 35–44 | 293 | 81.5 | 72.1 | 66.8 | 65.1 | 62.1 |
| 45–54 | 275 | 83.5 | 70.8 | 63.8 | 61.2 | 59.1 |
| 55–64 | 414 | 78.8 | 63.3 | 56.1 | 50.1 | 47.4 |
| 65–74 | 275 | 70.8 | 58.8 | 50.8 | 48.4 | 47.2 |
| 75 and over | 133 | 41.8 | 32.4 | 27.4 | 23.1 | 20.8 |
| $\chi^2$ for trend (1df) | | 94.5 | 80.3 | 88.7 | 96.7 | 98.7 |
| $P<$ | | 0.01 | 0.01 | 0.01 | 0.01 | 0.01 |
| $\chi^2$ for trend (1df) (controlling for treatment type[a]) | | 0.09 | 0.11 | 0.03 | 0.35 | 0.51 |
| $P<$ | | 0.80 | 0.80 | 0.90 | 0.70 | 0.46 |
| $\chi^2$ for trend (1df) (controlling for stage and treatment type) | | 0.03 | 0.49 | 0.10 | 0.01 | 0.03 |
| $P<$ | | 0.90 | 0.48 | 0.80 | 0.95 | 0.90 |

[a]Seven categories of treatment were controlled for, distinguishing between radical surgery and radiotherapy (alone or in combination), radiotherapy at different sites, and no treatment (including investigative surgery and palliative radiotherapy).

**Table VI** 5-year survival by socio-economic status (all patients)

| *Socio-economic status* | *Number entering study* | *Cumulative % surviving 1 year* | *2 years* | *3 years* | *4 years* | *5 years* |
|---|---|---|---|---|---|---|
| 1 Most affluent | 52 | 84.6 | 74.9 | 68.5 | 63.7 | 61.2 |
| 2 Affluent | 85 | 83.4 | 68.3 | 57.9 | 56.5 | 51.5 |
| 3 Above average | 229 | 80.8 | 67.5 | 58.5 | 55.8 | 55.2 |
| 4 Average | 363 | 76.9 | 61.9 | 58.1 | 54.6 | 52.4 |
| 5 Below average | 321 | 77.3 | 62.5 | 54.9 | 51.9 | 50.5 |
| 6 Deprived | 313 | 73.6 | 65.3 | 59.5 | 56.1 | 53.2 |
| 7 Most deprived | 225 | 73.2 | 62.9 | 57.5 | 53.1 | 51.9 |
| $\chi^2$ for trend (1df) | | 8.70 | 3.07 | 1.60 | 1.97 | 1.76 |
| $P<$ | | 0.01 | 0.08 | 0.21 | 0.17 | 0.19 |
| $\chi^2$ for trend (1df) (controlling for treatment type[a] | | 8.81 | 2.87 | 1.23 | 1.51 | 1.25 |
| $P<$ | | 0.01 | 0.10 | 0.26 | 0.22 | 0.26 |
| $\chi^2$ for trend (1df) (controlling for stage and treatment type) | | 2.15 | 0.02 | 0.11 | 0.06 | 0.11 |
| $P<$ | | 0.14 | 0.90 | 0.80 | 0.80 | 0.80 |

[a]Seven categories of treatment were controlled for, distinguishing between radical surgery and radiotherapy (alone or in combination), radiotherapy at different sites, and no treatment (including investigative surgery and palliative radiotherapy).

**Figure 4** 5-year survival by socio-economic status (Greater Glasgow health board area).

The incidence of invasive cancer of cervix in the West of Scotland varies considerably with socio-economic status. The disease is most common among women living in deprived areas. There is an almost threefold difference in incidence between the most, and least, affluent parts of the region. Two previous studies in Wales and the United States have shown similar differences in prevalence and in relative risk respectively (Sweetnam *et al.*, 1981; Fasal *et al.*, 1981). In this present study we have also shown that patients from less affluent areas who develop cancer of cervix are less likely to present with early stage disease (FIGO Stage I or II). Differences in stage at diagnosis accounted for almost all of the observed differences in survival by socio-economic status, both in Glasgow and in the West of Scotland as a whole. There is no evidence for a separate role for social status in determining survival independent of its relationship with tumour stage, as has been suggested elsewhere (Walker *et al.*, 1985).

A higher proportion of stage I tumours among patients living in the most affluent postcode sectors (deprivation

**Table VII** Cox Regression Analysis of 5-year survival by socio-economic status, age, stage, health board of residence, histology, tumour grade and year of treatment, controlling for treatment type[a] (all patients)[b,c]

| *Independent variable (risk group)* | | *Relative hazard* | *Significance (2-tailed P value <)* | *95 percent confidence limits* |
|---|---|---|---|---|
| *Socio-economic status* | | | | |
| | 1 Most affluent | 1.00 | | |
| | 2 Affluent | 0.965 | 0.910 | (0.525–1.774) |
| | 3 Above average | 1.165 | 0.576 | (0.681–1.993) |
| | 4 Average | 1.138 | 0.628 | (0.675–1.920) |
| | 5 Below average | 1.052 | 0.710 | (0.653–1.870) |
| | 6 Deprived | 0.914 | 0.738 | (0.539–1.549) |
| | 7 Most deprived | 1.111 | 0.706 | (0.643–1.920) |
| *Age* | Under 35 | 1.000 | | |
| | 35–44 | 1.103 | 0.578 | (0.780–1.559) |
| | 45–54 | 1.042 | 0.818 | (0.737–1.472) |
| | 55–64 | 0.993 | 0.966 | (0.720–1.369) |
| | 65–74 | 1.002 | 0.994 | (0.709–1.414) |
| | 75 and over | 1.141 | 0.506 | (0.773–1.684) |
| *Stage* | I | 1.000 | | |
| | II | 2.224 | 0.001 | (1.642–3.012) |
| | III | 4.593 | 0.001 | (3.402–6.201) |
| | IV | 7.808 | 0.001 | (5.584–10.92) |
| *Histology* | (Squamous) | 1.000 | | |
| | (Other) | 1.125 | 0.434 | (0.839–1.508) |
| *Tumour grade* | G1 | 1.000 | | |
| | G2 | 1.492 | 0.012 | (1.099–2.027) |
| | G3 | 1.628 | 0.002 | (1.209–2.193) |
| *Health Board* | GGHB | 1.000 | | |
| | Other | 1.020 | 0.826 | (0.853–1.221) |
| *Year of treatment* | 1980 | 1.000 | | |
| | 1981 | 0.975 | 0.872 | (0.716–1.327) |
| | 1982 | 0.878 | 0.434 | (0.634–1.215) |
| | 1983 | 0.981 | 0.910 | (0.704–1.368) |
| | 1984 | 0.815 | 0.220 | (0.588–1.130) |
| | 1985 | 0.818 | 0.256 | (0.578–1.157) |
| | 1986 | 0.997 | 0.984 | (0.726–1.367) |
| | 1987 | 0.861 | 0.376 | (0.618–1.200) |

[a]Seven categories of treatment were controlled for, distinguishing between radical surgery and radiotherapy (alone or in combination), radiotherapy at different sites, and no treatment (including investigative surgery and palliative radiotherapy).
[b]Six tumours were unstaged, 40 had no histology and 338 were ungraded. All of these cases were excluded from analysis.
[c]Analysis was carried out using program P2L of the Biomedical Data Package (BMDP) (Dixon, 1985).

categories 1–3) may reflect the success of screening initiatives in general practice within those sections of the community where the expectation of good health is high. Incidental take-up of screening in the course of ante-natal care is the likeliest explanation for a slightly above average proportion of stage I tumours among patients from the poorest areas of the West of Scotland (deprivation category 7). Most of these areas are located in the north and east of the city of Glasgow where birth rates are particularly high (Registrar General for Scotland, 1991). Older women are less likely to be screened incidentally by routine medical examination at family planning clinics or in the course of ante- or post-natal care, or to consult their doctor for gynaecological problems (Mamon *et al.*, 1990).

Well organised cervical screening programmes have brought about a considerable reduction in deaths from cervical cancer. Mortality has been reduced by the prompt detection and treatment both of pre-cancerous changes and of early stage cancer. These effects have been most striking in the Nordic countries (Laara *et al.*, 1987). In the UK the effects of screening have been less dramatic, but substantial reductions in mortality have followed the introduction of well conducted screening programmes in populations such as that of the Northeast of Scotland (MacGregor *et al.*, 1985; Duguid *et al.*, 1985).

A higher incidence of invasive cancer of cervix in the most disadvantaged areas may reflect lifestyle factors such as sexual behaviour and smoking but may also be due to a failure to detect pre-cancerous changes by screening. A recent evaluation of breast cancer screening in Edinburgh showed attendance rates of between 60 and 67% for practices in areas of high socio-economic status, compared with 45 to 54% for those in the poorest areas (Roberts *et al.*, 1990). Similarly, the tendency towards later stage at diagnosis among older, and to a lesser extent, poorer, patients suggests these individuals are not being screened effectively. These hypotheses are supported by the records of two general practices serving patients from very different socio-economic backgrounds. Out of a total of 2126 women aged between 23 and 70 and resident in Bearsden and Milngavie district, an affluent suburban area on the northwest edge of Glasgow (deprivation category 1), only 17% had no record of ever having a smear (personal communication, Dr Michael Kent, Milngavie General Practitioner). This figure compared with 46% of a similar number of women resident in the catchment area of an inner city health centre 5 miles to the southeast (deprivation categories 6 and 7) (personal communication, Prof J.H. Barber, University of Glasgow).

The cervical screening programme in the West of Scotland needs to reach that part of the female population most at risk. On the evidence of our study, this would include all those aged over 45, and in particular that section of this age group living in deprived areas.

The project reported in this paper was financed by Grant No.K/OPR/15/3/1/3 from the Scottish Home and Health Department. We are grateful to Vera Carstairs of the University of Edinburgh for making available unpublished data on deprivation by postcode sector, to the University of Glasgow Computing Service for data processing, and to Ann Graham and Pauline MacKinnon of the West of Scotland Cancer Surveillance Unit for cheking individual case records. We are also indebted to West of Scotland consultants in obstetrics and gynaecology for their permission to review patient records, and to the medical records officers at each hospital for sending those to us. Hospital discharge records were obtained (with the permission of the health boards concerned) from the Information and Statistics Division of The Scottish Health Service Common Services Agency. Our thanks are also due to Alison Jones and Archie Shanks of the Audiovisual Services Unit, Stobhill Hospital, Glasgow for computer graphics and preparation of illustrations.

### References

CARSTAIRS, V. & MORRIS, R. (1989). Deprivation: explaining differences in mortality between Scotland and England and Wales. *BMJ*, **299**, 886–889.

CARSTAIRS, V. & MORRIS, R. (1991). *Deprivation and Health in Scotland.* Aberdeen Univerity Press.

COOK, G.A. & DRAPER, G.J. (1984). Trends in cervical cancer and carcinoma in situ in Great Britain. *Br. J. Cancer*, **50**, 367–375.

COX, D.R. (1972). Regression models and life tables. *J. Roy. Statist. Soc.*, **34**, 187–220.

DIXON, W.J. (ed) (1985). *BMDP Statistical Software Manual.* Berkeley: University of California Press.

DUGUID, H.L.D., CURRIE, J. & DUNCAN, I.D. (1985). Screening for cervical intra-epithelial neoplasia in Dundee and Angus 1962-81 and its relation with invasive cervical cancer. *Lancet*, **i**, 1053–1056.

ELLIOTT, P.M., TATTERSALL, M.H.N., COPPLESON, M., RUSSELL, P., WONG, F., COATES, A.S., SOLOMON, H.J., BANNATYNE, P.M., ATKINSON, K.H. & MURRAY, J.C. (1989). Changing character of cervical cancer in young women. *BMJ*, **298**, 288–290.

FASAL, E., SIMMONS, M.E. & KAMPERT, J.B. (1981). Factors associated with high and low risk of cervical neoplasia. *J. Natl Cancer Inst.*, **66**, 631–636.

JUNOR, E.J., SYMONDS, R.P., WATSON, E.R. & LAMONT, D.W. (1989). Survival of younger cervical carcinoma patients treated by radical radiotherapy in the West of Scotland 1964–84. *Br. J. Obstet. Gynaecol.*, **96**, 522–528.

KOGEVINAS, M. (1990). Longitudinal Study: Socio-demographic differences in cancer survival. *OPCS Series LS* No 5. HMSO: London.

KOGEVINAS, M., MARMOT, M.G., FOX, A.J. & GOLDBLATT, P.O. (1991). Socioeconomic differences in cancer survival. *J. Epidemiol. Commun. Health*, **45**, 216–219.

LAARA, E., DAY, N.E. & HAKAMA, M. (1987). Trends in mortality from cervical cancer in the Nordic countries: association with organised screening programmes. *Lancet*, **i**, 1247–1249.

MACGREGOR, J.E., MOSS, S.M., PARKIN, D.M. & DAY, N.E. (1985). A case control study of cervical cancer screening in Northeast Scotland. *BMJ*, **290**, 1543–1546.

MAMON, J.A., SHEDIAC, M.C., CROSBY, C.B., SANDERS, B., MATANOSKI, G.M. & CELENTANO, D.D. (1990). Inner city women at risk for cervical cancer: behavioural and utilisation factors related to inadequate screening. *Prev Med.*, **19**, 363–376.

MEANWELL, C.A., KELLY, K.A., WILSON, S., ROGINSKI, C., WOODMAN, C., GRIFFITHS, R. & BLACKLEDGE, G. (1988). Young age as a prognostic factor in cervical cancer: analysis of population based data from 10022 cases. *BMJ*, **296**, 386–391.

MILNER, P.C. & WATTS, M. (1987). Effect of socioeconomic status on survival from cervical cancer in Sheffield. *J. Epidemiol. Commun. Health*, **41**, 200–203.

MURPHY, M., GOLDBLATT, P., THORNTON-JONES, H. & SILCOCKS, P. (1990). Survival among women with cancer of the uterine cervix: influence of marital status and social class. *J. Epidemiol. Commun. Health*, **44**, 293–296.

OSMOND, C., GARDNER, M.J., ACHESON, E.D. & ADELSTEIN, A.M. (1983). Trends in cancer mortality 1951–80, analyses by period of birth and death. *OPCS Series DH1* No 11. HMSO: London.

OSMOND, C., GARDNER, M.J. & ACHESON, E.D. (1982). Analysis of trends in cancer mortality in England and Wales during 1951–80 separating changes associated with period of birth and death. *BMJ*, **284**, 1005–1008.

PETO, R., PIKE, M.C., ARMITAGE, P., BRESLOW, N.E., COX, D.R., HOWARD, S.V., MANTEL, N., MCPHERSON, K., PETO, J. & SMITH, P.G. (1977). Design and analysis of randomised clinical trials requiring prolonged observations of each patient. II. Analysis and examples. *Br. J. Cancer*, **35**, 1–39.

PETTERSON, F. (ed) (1985). *19th Annual Report on the Results of Treatment in Gynaecological Cancer*. International Federation of Gynaecology and Obstetrics (FIGO): Stockholm.

REGISTRAR GENERAL FOR SCOTLAND. (1991). *Annual Report 1990*. General Register Office; Edinburgh.

ROBERTS, M.M., ALEXANDER, F.E., ANDERSON, T.J., CHETTY, U., DONNAN, P.T., FORREST, P., HEPBURN, W., HUGGINS, A., KIRKPATRICK, A.E., LAMB, J., MUIR, B.B. & PRESCOTT, R.J. (1990). Edinburgh trial of screening for breast cancer: mortality at seven years. *Lancet*, **i**, 241–246.

RUSSELL, J.M., BLAIR, V. & HUNTER, R.D. (1987). Cervical carinoma: prognosis in younger patients. *BMJ*, **295**, 300–303.

SWEETNAM, P., EVANS, D.M.D., HIBBARD, B.M. & JONES, J.M. (1981). The Cardiff cervical cytology study–prevalence and epidemiology of cervical neoplasia. *J. Epidemiol. Commun. Health*, **35**, 83–90.

VAGERO, D. & PERSSON, G. (1987). Cancer survival and social class in Sweden. *J. Epidemiol. Commun. Health*, **41**, 204–209.

WALKER, A.R.P., WALKER, B.F., SIWEDI, D., ISAACSON, C., VAN GELDEREN, C.J., ANDRONIKOU, A. & SEGAL, I. (1985). Low survival of South African urban black women with cervical cancer. *Br. J. Obstet. Gynaecol.*, **92**, 1272–1278.